# Investigation of Surface Behavior of DPPC and Curcumin in Langmuir Monolayers at the Air-Water Interface

**DOI:** 10.1155/2017/6582019

**Published:** 2017-11-07

**Authors:** Guoqing Xu, Changchun Hao, Lei Zhang, Runguang Sun

**Affiliations:** School of Physics and Information Technology, Shaanxi Normal University, Chang'an Street No. 199, Xi'an 710062, China

## Abstract

Langmuir monolayers of 1,2-dipalmitoyl-*sn*-glycero-3-phosphocholine (DPPC) and a mixture of DPPC with curcumin (CUR) have been investigated at the air-water interface through a combination of surface pressure measurements and atomic force microscopy (AFM) observation. By analyzing the correlation data of mean molecular areas, the compressibility coefficient, and other thermodynamic parameters, we obtained that the interaction between the two components perhaps was mainly governed by the hydrogen bonding between the amino group of DPPC and the hydroxyl groups of CUR. CUR markedly affected the surface compressibility, the thermodynamic stability, and the thermodynamic phase behaviors of mixed monolayers. The interaction between CUR and DPPC was sensitive to the components and the physical states of mixed monolayers under compression. Two-dimensional phase diagrams and interaction energies indicated that DPPC and CUR molecules were miscible in mixed monolayers. AFM images results were in agreement with these analyses results of experimental data. This study will encourage us to further research the application of CUR in the biomedical field.

## 1. Introduction

**Figure 1:**
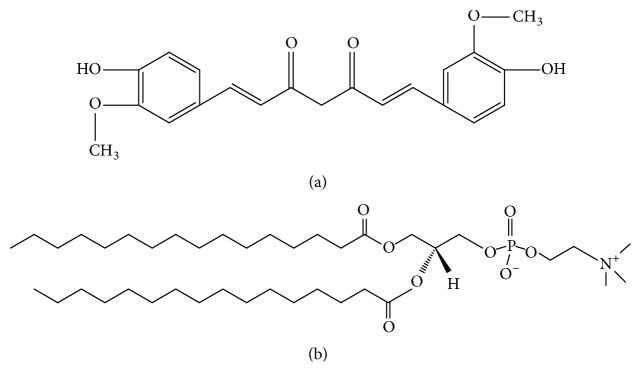
Molecular structures of (a) CUR and (b) DPPC.

Curcumin [1,7-bis(4-hydroxy-3-methoxyphenyl)-1,6-heptadiene-3,5-dione] (CUR) (Figure 1(a)) is one of the most common yellow colors in nature. It is obtained from the rhizomes of the plant* Curcuma longa* and other Zingiberaceae plants [1, 2]. CUR was usually used as a natural phenolic spice and food colorant a long time ago. It also was an important ingredient in curry and polyphenolic nutraceuticals in daily life [1, 3]. CUR belongs to acid polyphenolic compounds [4, 5]. Many previous studies proved that CUR has been widely investigated and was shown to have an important role in pharmacological activities because of its low toxicity, low adverse reactions, and special structure (hydroxyl groups of the benzene rings, the double bonds in the alkene part, and the diketone moiety) [6], such as anti-inflammatory, anticancer, antioxidant, anticoagulation, antiatherosclerotic, antimutagenic, antibiotic, antiviral, antifungal, and antiamyloid activities [6–8]. It has been reported that CUR can inhibit the proliferation and promote the apoptosis of many types of cancer cells, including lung cancer cells [9, 10]. But the interaction mechanisms between CUR and cancer cells are still unclear. 1,2-Dipalmitoyl-*sn*-glycero-3-phosphocholine (DPPC) (Figure 1(b)) is a major component in natural lung surfactants, also known as pulmonary surfactants [11, 12]. So, in our work, the Langmuir-Blodgett technology was used to investigate the interaction mechanisms between the interfacial monolayers of DPPC and CUR.

The behaviors of mixed DPPC-CUR monolayers with various mole fractions of CUR at the air-water interface have been investigated through the Langmuir-Blodgett (LB) technology and atomic force microscopy (AFM) observation. The miscibility of the two components, the thermodynamic stability of mixed monolayers, and the intermolecular interactions between DPPC and CUR molecules have been analyzed by the correlation data of surface pressure-mean molecular areas isotherms. In addition to these, the surface morphology features of mixed DPPC-CUR monolayers were observed with AFM. This research will help us obtain an insight into the biological activity of CUR in the biomedical field.

## 2. Experimental Details

### 2.1. Materials

1,2-Dipalmitoyl-*sn*-glycero-3-phosphocholine (DPPC) and curcumin (CUR) were purchased from Sigma-Aldrich Chemical Company, and both of them were used without further purification. DPPC and CUR were dissolved in chloroform/methanol (3 : 1, v/v) mixture and methanol solution (due to the low solubility of CUR in water) at a concentration of 0.1 mg/mL, respectively. Chloroform and methanol were also purchased from Sigma-Aldrich Chemical Company. For all the experiments, ultrapure water (resistivity = 18 MΩ cm) was employed for the subphase and cleaning the trough.

### 2.2. *π*-*A* Isotherms of Mixed Monolayers

Certain volumes of the two solutions were dropped onto the surface of the subphase with a Hamilton microsyringe. The mixed DPPC-CUR monolayers with different mole fractions of CUR were obtained using a computer-controlled commercial device (Minitrough, KSV, Helsinki, Finland) with two symmetric moving barriers at a constant rate of 1 mm/min [13, 14]. After 15 min of evaporating the organic solutions and equilibrating the monolayer, the barriers were compressed. The Wilhelmy plate technique would help us record the surface pressure accurately, and these data were simultaneously recorded by the computer [15, 16]. In addition, the Langmuir-Blodgett technique was used to transfer mixed DPPC-CUR monolayers onto freshly cleaved micas at the surface pressure of 15 mN/m with a vertical pulling method at a constant transfer velocity of 0.5 mm/min, which was used as a one-layer LB film for atomic force microscopy (AFM) observation [17]. Every experimental test was repeated at least three times to obtain good reproducibility. All measurements were carried out at the temperature of 291 ± 1 K.

### 2.3. Atomic Force Microscopy (AFM) Observation

The surface morphology features of mixed DPPC-CUR monolayers with different mole fractions of CUR were directly visualized by using an SPM-9500-J3 AFM (Shimadzu Corporation, Japan) in the tapping mode. The AFM images (512 × 512 points per line) in height mode were collected at a scanning rate of 1.0 Hz using a micro-V-shaped cantilever probe (Olympus Corporation, Japan). The nominal spring constant of the probe was 0.06 N/m. The probe was made of Si_3_N_4_ [13, 18]. All measurements were carried out at the temperature of 291 ± 1 K.

## 3. Results and Discussion

### 3.1. *π*-*A* Isotherms at Discrete Mole Fractions at Air-Water Interface

**Figure 2:**
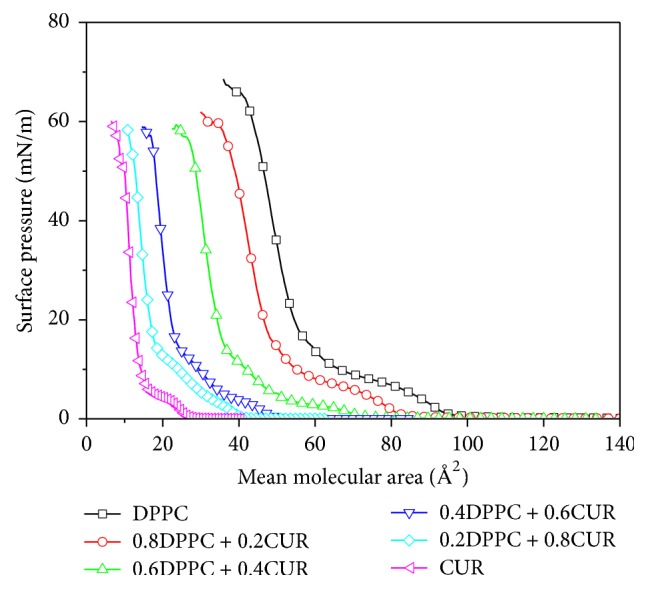
Surface pressure-area (*π*-*A*) isotherms of mixed DPPC-CUR monolayers.

The surface pressure (*π*)-mean molecular area (*A*) isotherms of mixed DPPC/CUR monolayers with various mole fractions of CUR (*X*_CUR_ = 0, 0.2, 0.4, 0.6, 0.8, and 1) at the air-water interface are shown in Figure 2. The isotherm of pure DPPC monolayer showed its inherent characteristics; for example, there was a main transition at ~8 mN/m, and the collapse surface pressure was ~65 mN/m. All of them were consistent with the reported literature [19, 20]. From Figure 2, we observed that the collapse surface pressure was ~50 mN/m for pure CUR. The collapse pressure of pure CUR was obviously lower than that of DPPC. The reason perhaps was that the DPPC molecule has a bigger headgroup with two hydrophobic tail chains, while the structure of the CUR molecule is symmetrical. From Figure 2, we could also obtain that the addition of CUR made the curves move towards the direction of the smaller mean molecular area and the lift-off molecular area gradually decreased at the same time. The isotherms of the mixed system appeared in the order between those of the two pure monolayers. The shape of isotherms arrayed systematically and the slopes decreased with the increase of *X*_CUR_ (the values of the slopes were 209.86, 189.114, 182.85, 167.80, 131.34, and 119.33, resp., with the increase of *X*_CUR_ from 0 to 1). The influence of CUR on DPPC monolayers was caused by the interaction between DPPC and CUR molecules.

### 3.2. Miscibility of the Mixed Monolayers

In order to ensure the miscibility of the two components, we calculated the ideal values of the molecular area (*A*_id_) of the mixed DPPC-CUR monolayers. *A*_id_ was calculated from the following equation [21]:

(1) $$\begin{array}{c} A_{id}=A_{1}X_{1}+A_{2}X_{2}, \end{array}$$

where *A*_1_ and *A*_2_ are the molecular areas of components 1 and 2 at a definite surface pressure. *X*_1_ and *X*_2_ are the mole fractions of components 1 and 2 in mixed monolayers. The information of the miscibility can be obtained by comparing the deviation between the experimental mean molecular areas (*A*_exp_) and *A*_id_. If two components are immiscible or ideally miscible but do not interact, the curve of the mean molecular areas is a straight line. On the contrary, if the curve exhibits nonlinear characteristics, it indicates that the two components are miscible in the mixed monolayer [22].

**Figure 3:**
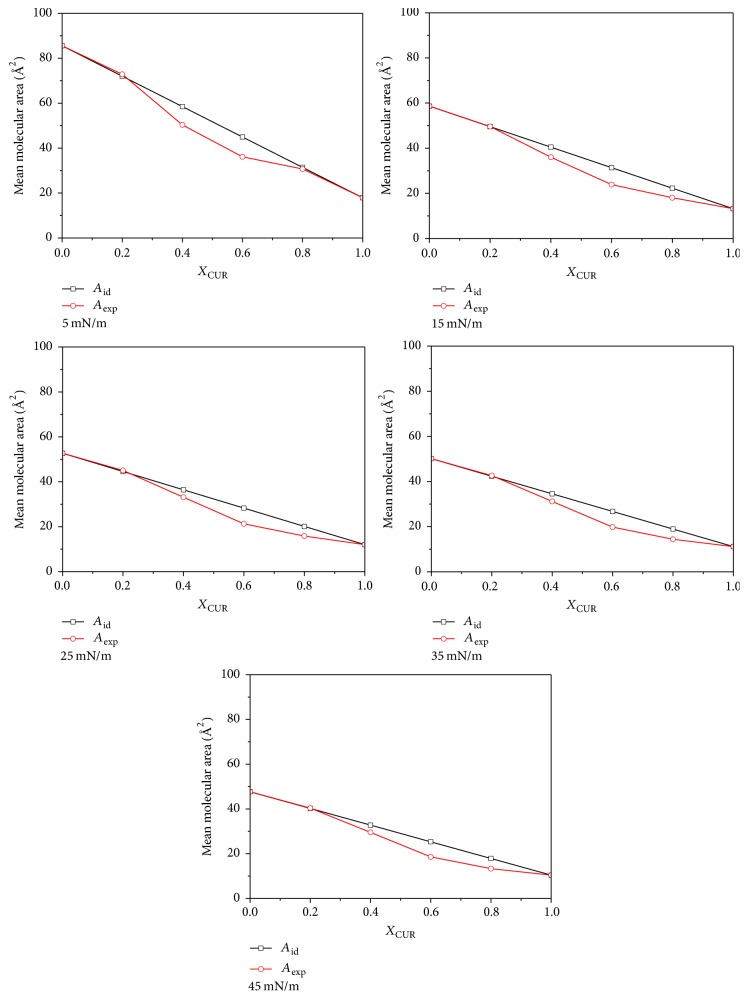
Mean molecular area as a function of the mole fraction of CUR in the mixed DPPC-CUR monolayers on water subphase at different values of surface pressures (*π* = 5, 15, 25, 35, and 45 mN/m).

The mean molecular areas (*A*_exp_ and *A*_id_) as a function of the mole fraction of CUR (*X*_CUR_ = 0, 0.2, 0.4, 0.6, 0.8, and 1) at different surface pressures (*π* = 5, 15, 25, 35, and 45 mN/m) are shown in Figure 3. As can be seen from Figure 3, the curves of *A*_exp_ exhibited nonlinear characteristics for different surface pressures. It indicated that DPPC/CUR were considered to be miscible and interacted with each other at the air-water interface. From Figure 3, we also obtained that the experimental data were almost in accord with the theoretical ones at *X*_CUR_ = 0.2 for all different surface pressures. This indicated that the two compositions mixed may be near ideality. However, the negative deviations from the ideal mixing lines were observed when *X*_CUR_ ≥ 0.4. This indicated that the two components were miscible easily. These results suggested that the state of mixed monolayers may be divided at least into two parts above and below the mole fraction of 0.4, and the interaction mechanisms were associated with the composition of monolayers.

The analysis of the excess mean molecular area (Δ*A*_exc_) is an accurate way to study the miscibility of the two components and the different intermolecular interactions between the two components in the mixed monolayers. The Δ*A*_exc_ value at a given surface pressure can be calculated by the equation [23]

(2) $$\begin{array}{c} \Delta A_{exc}=A_{exp}-A_{id}. \end{array}$$

When Δ*A*_exc_ = 0, this means the two components are completely immiscible or perfectly miscible. When Δ*A*_exc_ ≠ 0, this suggests the miscibility of the two components and different interaction mechanisms occur in the mixed monolayers [24].

**Figure 4:**
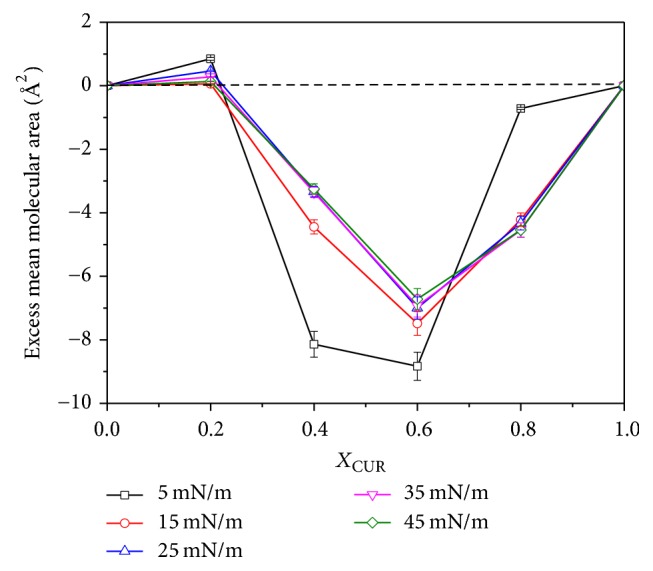
The excess area (Δ*A*_exc_) as a function of *X*_CUR_ at different surface pressures (*π* = 5, 15, 25, 35, and 45 mN/m).

The Δ*A*_exc_ values as a function of the mole fraction of CUR (*X*_CUR_ = 0, 0.2, 0.4, 0.6, 0.8, and 1) at different surface pressures (*π* = 5, 15, 25, 35, and 45 mN/m) are shown in Figure 4. We found that, for all different surface pressures, the Δ*A*_exc_ values were positive at *X*_CUR_ = 0.2 and negative at *X*_CUR_ ≥ 0.4 within the limit of error. This indicated that, at low mole fraction of CUR (*X*_CUR_ = 0.2), the interactions between like molecules (DPPC-DPPC and CUR-CUR) were stronger than that between DPPC and CUR, which meant that the two components may be miscible difficultly. With the increase of *X*_CUR_, the interaction between DPPC and CUR molecules in the mixed monolayer was more attractive than that between the molecules in their respective one-component monolayers and the two components were miscible easily at the interface, which resulted in the decrease of the mean molecular areas of the mixed monolayers. The attractive interaction between the two components perhaps was mainly governed by the hydrogen bonding between the amino group of DPPC and the hydroxyl groups of CUR.

The negative value of Δ*A*_exc_ means the presence of CUR molecules had a contraction effect on the phospholipid monolayers at the range of 0.4 ≤ *X*_CUR_ ≤ 0.8. With the increase of the surface pressure, the absolute values of Δ*A*_exc_ decreased except for the case of *X*_CUR_ = 0.8 at *π* = 5 mN/m. When *X*_CUR_ = 0.6, the Δ*A*_exc_ values obtained the minimum values for all different surface pressures. This meant that the interaction between DPPC and CUR molecules was strongest at *X*_CUR_ = 0.6 for the same surface pressure. These results suggested that the interaction between DPPC and CUR molecules and the intensity of the contraction effect of CUR on the phospholipid monolayer were associated with the composition of monolayers and the surface pressure.

In order to study the intensity of the contraction effect of CUR on the phospholipid monolayer exactly, the percent of condensation (*C*%) of the mixed monolayer was used to evaluate the intensity of the contraction effect. *C*% at a given surface pressure can be calculated by the following equation [25, 26]:

(3) $$\begin{array}{c} C\%=\left(\;\frac{A_{id}-A_{exp}}{A_{id}}\;\right)\times 100\%. \end{array}$$

The negative and positive values of *C*% mean the expansion and contraction effect caused by CUR, respectively. The higher absolute value of *C*% represents the stronger expansion or condensation effect [27]. The data of the mixed DPPC-CUR monolayers at different surface pressures (*π* = 5, 15, 25, 35, and 45 mN/m) are presented in Table 1.

As can be seen from Table 1, when *X*_CUR_ = 0.2, the *C*% values were negative for all different surface pressures, which meant the expansion effect caused by CUR, and the *C*% value got the minimum (*C*% = −1.175%) at the surface pressure of 5 mN/m. With the increase of surface pressure, the *C*% values at *X*_CUR_ = 0.2 increased to −0.333%. This indicated that, with the increase of surface pressure, the two compositions mixed may be near ideality in the ordered-tilted condensed state. The *C*% values were positive except for the case of *X*_CUR_ = 0.2. Another interesting thing observed in Table 1 was that the *C*% values at *X*_CUR_ = 0.6 were higher than that at other cases at the same surface pressure and got the maximum value (*C*% = 26.534%) at the surface pressure of 45 mN/m. This indicated that when *X*_CUR_ = 0.6, the intensity of the contraction effect reached the extremum at *π* = 45 mN/m. The reason perhaps was that the attractive interaction between the two components was enhanced at higher surface pressure. The expansion and condensation effects were sensitive to the physical state of monolayers and the compositions of mixed monolayers.

### 3.3. Compressibility Analysis

The compressibility coefficient (*C*_*S*_^−1^) obtained from *π*-*A* isotherms is a useful parameter to characterize the compression elasticity and phase transition behaviors of the monolayers at the air-water interface under compression [12, 28]. *C*_*S*_^−1^ can be calculated by the following equation:

(4) $$\begin{array}{c} C_{S}^{-1}=-A{\left(\;\frac{\partial \pi }{\partial A}\;\right)}_{T}, \end{array}$$

where *A* represents the mean molecular area and *π* represents the surface pressure. In general, the higher *C*_*S*_^−1^ value means the monolayer is difficult to compress [29]. According to the early studies by Davies and Rideal [30], the compressibility coefficient (*C*_*S*_^−1^) is a useful parameter to quantify the physical states of monolayers. The classification of the physical states of monolayers is shown as follows: gas (G) phase (*C*_*S*_^−1^ < 12.5 mN/m), liquid expansion (LE) phase (*C*_*S*_^−1^: 12.5–50 mN/m), liquid (liquid expansion/liquid condensed coexistence (LE/LC)) phase (*C*_*S*_^−1^: 50–100 mN/m), liquid condensed (LC) phase (*C*_*S*_^−1^: 100–250 mN/m), and condensed (C) phase (*C*_*S*_^−1^ > 250 mN/m) [29, 30]. The minima of *C*_*S*_^−1^ correspond to the phase transition point of lipid monolayers [13].

**Figure 5:**
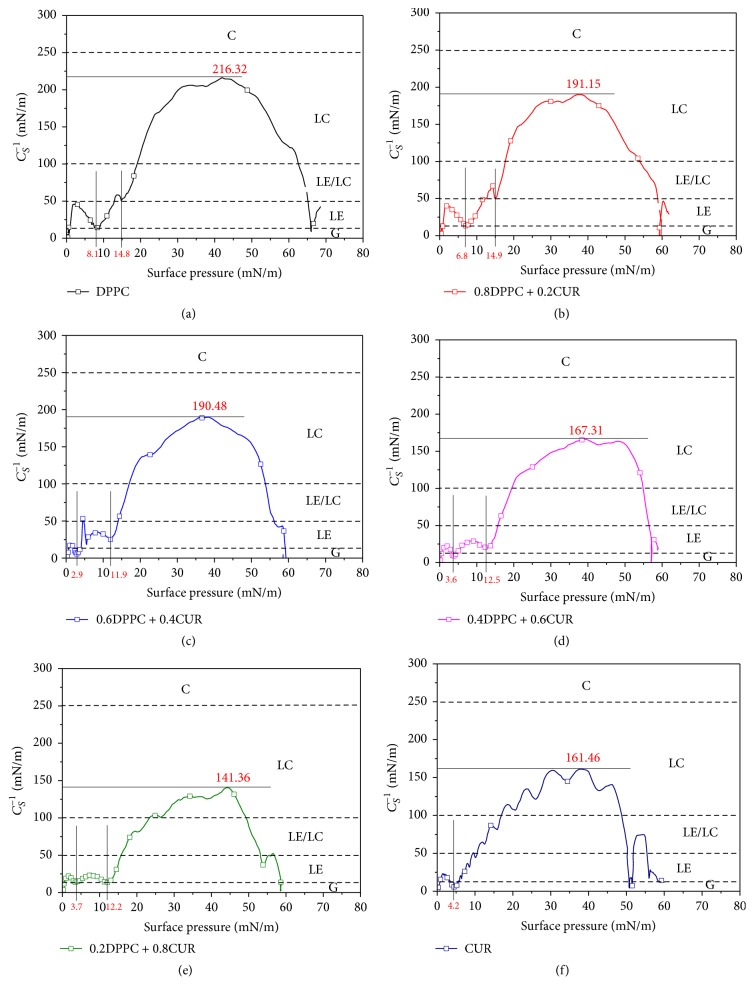
The surface compression elasticity of mixed DPPC-CUR monolayers as a function of surface pressure for discrete *X*_CUR_.

The compression elasticity-surface pressure (*C*_*S*_^−1^-*π*) curves obtained from *π*-*A* isotherms are presented in Figure 5. We could see that the maximum of *C*_*S*_^−1^ of the pure DPPC monolayer was 216.32 mN/m and two minimum values on the curve were observed at the surface pressures of ~8 mN/m and ~15 mN/m (Figure 5(a)), which corresponded to the phase transitions from liquid expansion (LE) to liquid expansion (LE)/liquid condensed (LC) coexistence phase and LE/LC to LC phase, respectively. The phase transition point of LE to LE/LC phase moved towards the direction of lower surface pressure with the increase of *X*_CUR_ (up to 0.2) (Figures 5(a) and 5(b)). When *X*_CUR_ ≥ 0.4, the phase transition point from LE/LC to LC phase disappeared. When 0.4 ≤ *X*_CUR_ ≤ 0.8, two minimum values were observed on each curve, which corresponded to the phase transitions from gas (G) to LE phase and LE to LE/LC phase, respectively (Figures 5(c), 5(d), and 5(e)). In the case of CUR alone, we found that there was only a minimum value at ~4 mN/m (Figure 5(f)). This indicated that the phase transition from G to LE phase occurred under compression. The phase transition points of mixed monolayers from G to LE phase moved towards the direction of higher surface pressure with the increase of *X*_CUR_. These results also indicated that the mixed monolayer state was divided into two parts above and below the mole fraction of 0.4. When *X*_CUR_ < 0.4, the isotherms of mixed monolayers followed the pattern of pure DPPC monolayer, while followed the pattern of CUR when *X*_CUR_ ≥ 0.4. In addition, the maximum value of *C*_*S*_^−1^ (*C*_*S*_^−1^_max_) decreased from 216.32 to 140.56 mN/m with the mole fraction of CUR increasing from 0 to 0.8. This indicated that the addition of CUR to the lipid monolayers made the monolayers more disordered, and the compressibility of monolayers gradually increased. It was also worth noting that the *C*_*S*_^−1^_max_ value was 161.46 mN/m for pure CUR monolayer, which was higher than the case of *X*_CUR_ = 0.8. These results obtained from *C*_*S*_^−1^-*π* curves indicated that the compression elasticity and phase transition behaviors of mixed monolayers were closely related to the interaction between DPPC and CUR molecules.

### 3.4. Thermodynamic Stability Analysis of the Binary Monolayers

The excess Gibbs free energy (Δ*G*_ex_) was used to quantitatively analyze the information of the thermodynamic stability of mixed monolayers; Δ*G*_ex_ can be calculated from the following equation [31, 32]:

(5) $$\begin{array}{c} \Delta G_{ex}=\overset{\pi }{\underset{0}{\int }}\left[\;A_{exp}-\left(\;X_{1}A_{1}+X_{2}A_{2}\;\right)\;\right]d\pi , \end{array}$$

where *A*_exp_ represents the experimental mean molecular area. *A*_1_ and *A*_2_ denote the mean molecular areas of components 1 and 2 at a definite surface pressure, respectively.* X*_1_ and* X*_2_ are their mole fractions of components 1 and 2 in mixed monolayers. *π* is the surface pressure of monolayers. If Δ*G*_ex_ = 0, this means the two components are ideally mixed or totally immiscible. The negative value of this parameter means that the two components are miscible easily at the interface and the attractive interaction between the two molecules makes the mixed monolayers stable. On the contrary, the positive value means the mixed monolayers have lower thermodynamic stability [23, 33]. The minimum of Δ*G*_ex_ indicates the highest thermodynamic stability of the mixed monolayer in comparison with other monolayers.

**Figure 6:**
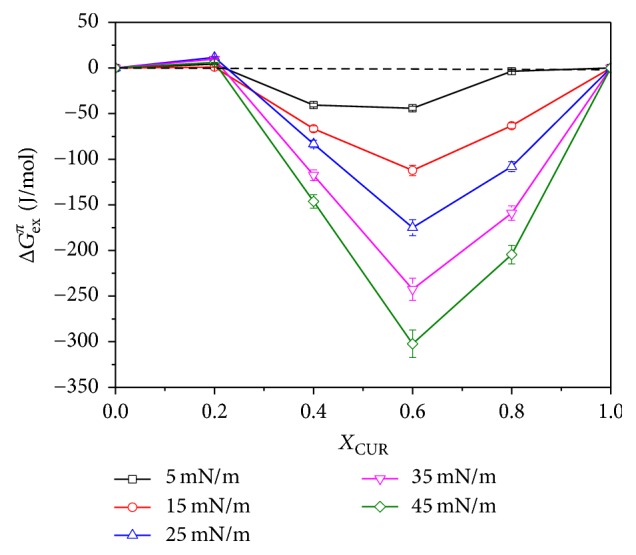
The excess Gibbs free energy (Δ*G*_ex_) values of the mixed DPPC-CUR monolayers at the air-water interface at discrete surface pressures.

The Δ*G*_ex_ values as a function of the mole fraction of CUR (*X*_CUR_ = 0, 0.2, 0.4, 0.6, 0.8, and 1) at a series of discrete surface pressures are presented in Figure 6. Positive values were obtained in the case of *X*_CUR_ = 0.2 for all different surface pressures, and Δ*G*_ex_ values increased with the increase of surface pressure. This indicated that the mixed monolayers had low thermodynamic stability. The Δ*G*_ex_ values were all negative at the range of 0.4 ≤ *X*_CUR_ ≤ 0.8 and became more negative with the increase of surface pressure. This also indicated that the increase of surface pressure resulted in the enhancement of the attractive interactions between DPPC and CUR molecules. The thermodynamic stability of the mixed monolayers was higher than that of pure monolayers and the mixed monolayer of *X*_CUR_ = 0.2. Attention should be paid to the case of *X*_CUR_ = 0.6; the minimums of Δ*G*_ex_ were got at the same surface pressure for all mixtures. This revealed that the hydrogen bonding between the two components was strongest at *X*_CUR_ = 0.6, which made the mixed monolayer have the highest thermodynamic stability.

The regular solution theory (RST) was applied to further analyze the thermodynamic information of the mixed monolayers in more detail [34]. From the values of Δ*G*_ex_, the interaction parameter *ξ* and activity coefficients *γ*_*i*_ of DPPC and CUR at a given surface pressure can be calculated by the following equations [23, 34, 35]:

(6) ξ=ΔGexRTX1X22+X2X12=ΔGexRTX1X2,ln⁡γ1=ξX22,ln⁡γ2=ξX12,

where *R* is the Boltzmann constant, *T* is the absolute temperature, and *X*_1_ and *X*_2_ denote the mole fractions of components 1 and 2 in the mixed film. The interaction parameter *ξ* is a measurement of the cohesive forces between different molecules [36, 37]. The negative value of *ξ* denotes a stronger attractive interaction between the two molecules while the positive value of *ξ* means a stronger repulsive interaction between like molecules [38]. The bigger absolute value of *ξ* means the stronger interaction between molecules.

**Figure 7:**
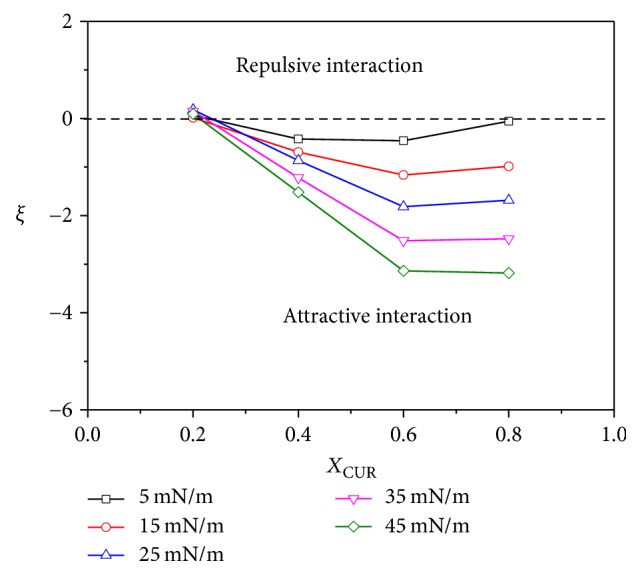
Interaction parameter (*ξ*) of DPPC-CUR monolayers versus *X*_CUR_ at discrete surface pressures.

**Figure 8:**
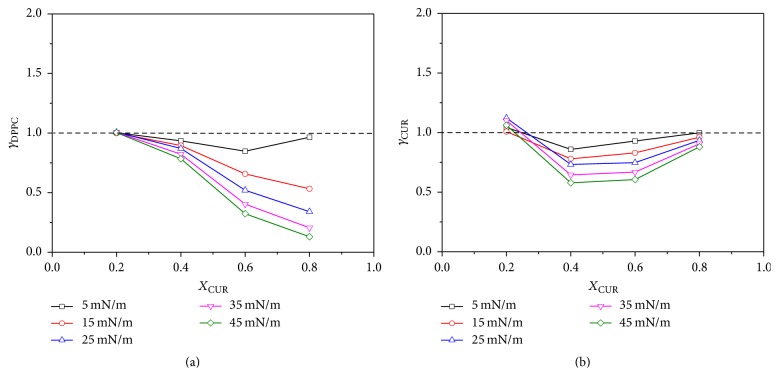
Activity coefficients (a) *γ*_DPPC_ and (b) *γ*_CUR_ of mixed DPPC-CUR monolayers versus *X*_CUR_ at discrete surface pressures.

The interaction parameter *ξ* and the activity coefficients *γ*_*i*_ of DPPC and CUR as a function of *X*_CUR_ at different surface pressures are shown in Figures 7 and 8, respectively. From Figure 7, we could see that, for all pressures, the *ξ* values were all positive at *X*_CUR_ = 0.2 and all negative at 0.4 ≤ *X*_CUR_ ≤ 0.8, respectively. The positive values at *X*_CUR_ = 0.2 suggested that the repulsive interactions between like molecules (DPPC-DPPC and CUR-CUR) were stronger than that between DPPC and CUR molecules in the mixed monolayer, which resulted in the low thermodynamic stability of the mixed monolayer. However, the negative values at the range of 0.4 ≤ *X*_CUR_ ≤ 0.8 indicated that the interaction between DPPC and CUR in mixed monolayers became more strongly attractive compared with the interactions between like molecules (DPPC-DPPC and CUR-CUR) in their respective one-component monolayers. Another interesting thing observed in Figure 7 was that, at the same *X*_CUR_, the absolute values of *ξ* increased with the increase of surface pressure. This also indicated that the interaction between molecules became stronger in the ordered-tilted condensed state. In addition, the absolute value of *ξ* at *X*_CUR_ = 0.6 was the highest for all mixtures at the surface pressure of 45 mN/m. This suggested that when *X*_CUR_ = 0.6, DPPC could interact most attractively with CUR in the ordered-tilted condensed state. These results were consistent with the above analysis. This situation could also be reflected by the activity coefficients. In general, if two molecules are noninteracting, surface activity coefficients will be equal to unity (*γ*_1_ = *γ*_2_ = 1) [39]. From Figures 8(a) and 8(b), we could observe that *γ*_DPPC_ values were very close to one (unity) at *X*_CUR_ = 0.2 and then markedly decreased with the increase of *X*_CUR_ from 0.4 to 0.8 for the same surface pressure. However, the *γ*_CUR_ values decreased to a minimum value (up to *X*_CUR_ = 0.4) and then increased with the increase of *X*_CUR_. At *X*_CUR_ = 0.8, *γ*_CUR_ was almost equal to one (unity) for the same surface pressure. The values of *γ*_DPPC_ and *γ*_CUR_ decreased with the increase of surface pressure for all *X*_CUR_, which meant that the intermolecular interactions between DPPC and CUR strengthen with the improvement of surface pressure. An ordered-tilted condensed state provided a better interaction environment. For the cases of *X*_CUR_ = 0.6 and 0.8, *γ*_DPPC_ = 0.32 and 0.13 while *γ*_CUR_ = 0.61 and 0.88 at the surface pressure of 45 mN/m, respectively. This revealed that DPPC and CUR exhibited an attractive interaction with each other, especially at *X*_CUR_ = 0.6. DPPC molecules (as the minority) could most attractively interact with CUR molecules (as the majority) at *X*_CUR_ = 0.8 compared with single component monolayer.

### 3.5. Two-Dimensional Phase Diagrams

**Figure 9:**
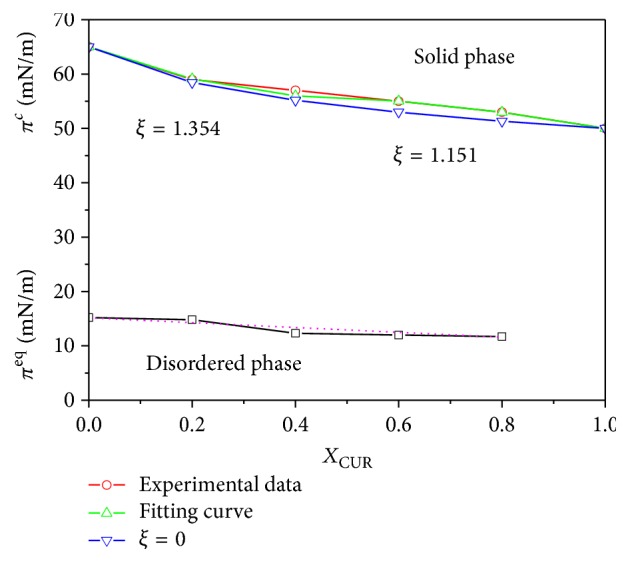
Two-dimensional phase diagrams based on the variation in phase transition pressure (*π*^eq^) and collapse pressure (*π*^*c*^) on pure water subphase at 291 ± 1 K as a function of *X*_CUR_. The pink line was calculated according to (7) for *ξ* = 0. The black and red lines represent experimental *π*^eq^ and *π*^*c*^ values, respectively. The blue line was calculated using (7) and was made coincident with the experimental values by adjusting *ξ*.

The two-dimensional phase diagram is a significative method to learn the thermodynamic information related to the phase behavior of the Langmuir monolayer. The two-dimensional phase diagrams of two-dimensional systems are constructed by using the data of the disordered/ordered transition pressure (*π*^eq^) and the monolayer collapse pressure (*π*^*c*^) obtained from the *π*-*A* isotherms [39, 40]. The changes of the phase diagrams of DPPC-CUR system at various molar fractions of CUR are shown in Figure 9. The linear distribution of *π*^eq^ with the change of molar fractions of CUR indicated that DPPC and CUR molecules were miscible in the mixed monolayers [18]. Under the assumption of a regular surface mixture, the following Joos equation can theoretically simulate the coexistence phase boundary between ordered monolayer (2D phase) and bulk phases (3D phase) of molecules spread on the surface [39–41]:

(7) 1=X1eπc,m−πc,1/KTω1eξX12+X2eπc,m−πc,2/KTω2eξX22,

where *X*_1_ and *X*_2_ denote the mole fractions of components 1 and 2 in the given binary mixed monolayers, respectively. *π*_*c*,1_ and *π*_*c*,2_ represent the corresponding collapse pressures of components 1 and 2. *π*_*c*,*m*_ represents the collapse pressure of the mixed monolayer at a given composition of *X*_1_ and *X*_2_. *ω*_1_ and *ω*_2_ are the corresponding limiting molecular areas of components 1 and 2 at the collapse points, respectively. *ξ* is the interaction parameter. *K* and *T* are the Boltzmann constant and the temperature in Kelvin, respectively. The interaction parameter *ξ* can be used to obtain the interaction energy (−Δ*ε*) [35, 42]:

(8) $$\begin{array}{c} -\Delta \varepsilon =-\frac{\xi RT}{z}, \end{array}$$

where *z* is the number of nearest neighbors (equal to 6) in a hexagonal close-packed monolayer.

As can be seen from Figure 9, the phase behaviors of the mixed DPPC-CUR monolayers can be divided into two parts: *ξ* = 1.354 for 0 ≤ *X*_CUR_ ≤ 0.2 and *ξ* = 1.151 for 0.2 < *X*_CUR_ ≤ 1. Their interaction energies (−Δ*ε*) were −545.55 J/mol and −463.76 J/mol, respectively. When −Δ*ε* < 2 *RT* (4958.7 J/mol), the two components are miscible in mixed monolayers [43]. So, DPPC and CUR molecules were miscible in the mixed monolayers for all various mole ratios.

### 3.6. AFM Observation

**Figure 10:**
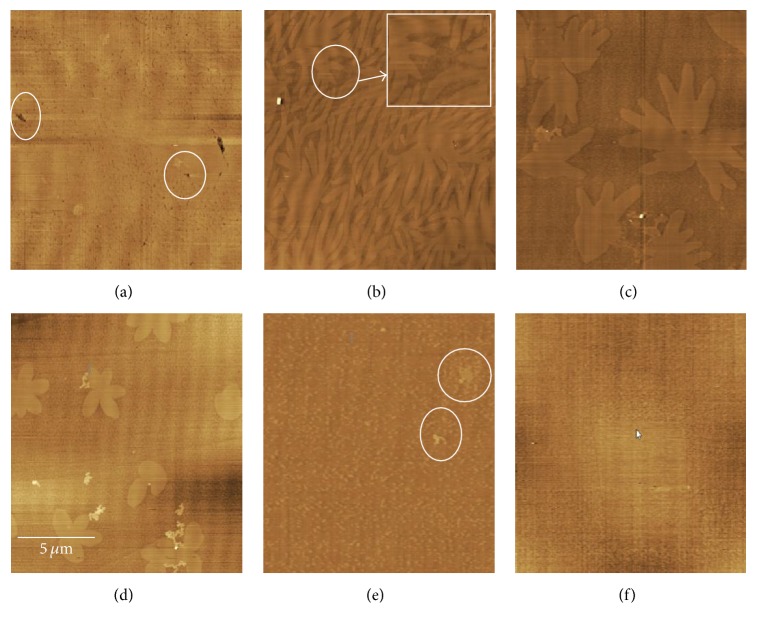
AFM images of the DPPC-CUR monolayers for (a) *X*_CUR_ = 0 (DPPC), (b) *X*_CUR_ = 0.2, (c) *X*_CUR_ = 0.4, (d) *X*_CUR_ = 0.6, (e) *X*_CUR_ = 0.8, and (f) *X*_CUR_ = 1 (CUR) at the surface pressure of 15 mN/m. Scanning range: 15 *μ*m × 15 *μ*m.

The monolayers were transferred onto mica substrates at the surface pressure of 15 mN/m, and atomic force microscopy was used to image the topography of monolayers at the nanoscale level. The AFM images provide more information about the molecular interactions, the miscibility of the two components, domain growth, and phase separation of monolayers at the air-water interface [38, 40]. AFM images of mixed DPPC-CUR monolayers with the six different mole fractions are shown in Figures 10(a)–10(f). The structures of the lipid monolayers had changed a lot with the increase of *X*_CUR_. The observed domain of pure DPPC monolayer showed a uniform pattern with a mass of compact platforms and relatively fewer pore-like structures (Figure 10(a)). When *X*_CUR_ = 0.2, some platforms in the shape of different branches could be seen in the image (Figure 10(b)). The interactions between DPPC and CUR molecules made the structure of the platform become small. As can be seen from the magnified area in Figure 10(b), a variety of microdomains of complexes appeared in the observed domain. The *ξ* and Δ*G*_ex_ values were positive at *X*_CUR_ = 0.2 at the surface pressure of 15 mN/m. This indicated that the monolayer had poor stability. These results were consistent with the observation of the AFM image. When *X*_CUR_ = 0.4, the branch-like structures of lipid monolayers changed into floriated platform structures, and more microdomains of complexes appeared in the observed image (Figure 10(c)). Compared with the case of *X*_CUR_ = 0.4, the observed AFM image showed that the floriated platform structures changed into smaller microdomains of complexes when *X*_CUR_ = 0.6 (Figure 10(d)). When *X*_CUR_ = 0.8, the floriated platform structures almost disappeared and more and more microdomains of complexes appeared in the observed domain (Figure 10(e)). This indicated that the thermodynamic stability of the mixed monolayer at *X*_CUR_ = 0.8 was less than that at *X*_CUR_ = 0.6. These results revealed that CUR molecules had a contraction effect on the DPPC lipid monolayer. For the case of *X*_CUR_ = 1, from the observed image, we observed that there were no obvious membrane structures (Figure 10(f)), which may be caused by the structural property of CUR molecule. From Figures 10(a)–10(e), it was obtained that DPPC and CUR molecules were miscible in the mixed monolayers.

**Figure 11:**
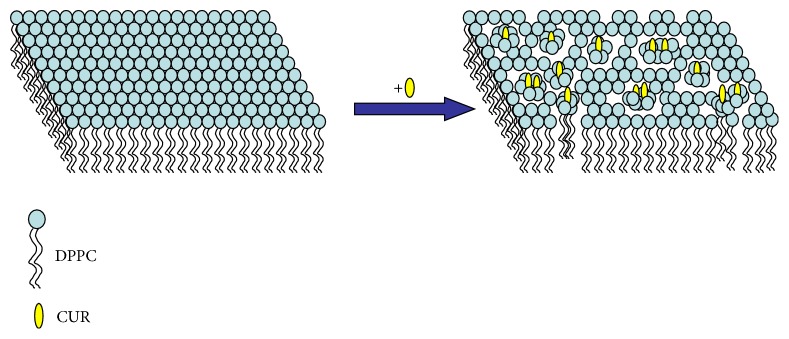
The model of the contraction effect of CUR molecules on the DPPC lipid monolayer.

CUR has been widely investigated as an important role in the pharmacological activities because of its low toxicity, low adverse reactions, and special structure (hydroxyl groups of the benzene rings, the double bonds in the alkene part, and the diketone moiety). DPPC is a major component in natural lung surfactants. In our work, the experimental results indicated that CUR has an expansion or contraction effect on DPPC monolayers. In addition, CUR markedly affected the compressibility, the thermodynamic stability, and the thermodynamic phase behaviors of the mixed monolayers. At *X*_CUR_ = 0.2, the interactions between like molecules (DPPC-DPPC and CUR-CUR) were stronger than that between DPPC-CUR. With the increase of surface pressure, the two compositions mixed may be near ideality in the ordered-tilted condensed state. At 0.4 ≤ *X*_CUR_ ≤ 1, the interaction between DPPC and CUR molecules in the mixed film was more attractive than that between the molecules in their respective one-component monolayers, and the two components were partially miscible at the interface. The reason was that the interaction between the two components was mainly governed by the hydrogen bonding between the amino group of DPPC and the hydroxyl groups of CUR. When *X*_CUR_ = 0.6, the strongest attractive interaction between CUR and DPPC was obtained at the surface pressure of 45 mN/m. This indicated that the interaction mechanism between CUR and DPPC molecules was sensitive to the components and the physical states of the mixed monolayers under compression. A similar behavior was obtained in DPPC/resveratrol monolayers [44]. In Hoda et al.'s work, they also obtained that the interaction ways were sensitive to the physical states of lipid monolayers [41]. When 0.4 ≤ *X*_CUR_ ≤ 1, the attractive interaction made the thermodynamic stability of the mixed films higher than that of the pure DPPC monolayer and the mixed monolayer of *X*_CUR_ = 0.2. The model of the contraction effect of CUR molecules on DPPC lipid monolayer is shown in Figure 11. The contraction effect made some defect structures occur in the DPPC monolayer. The study provides an important experimental basis and theoretical support for learning the interaction mechanism between DPPC and CUR molecules and getting an insight into the biological activity of CUR in the biomedical field.

## 4. Conclusion

In this work, the interaction between CUR and DPPC molecules at the air-water interface has been studied by analyzing the miscibility, the thermodynamic stability, the morphology structure, and the two-dimensional phase diagram of the mixed DPPC-CUR monolayers at different mole ratios. It was obtained that the interaction between CUR and DPPC molecules depends on the components of the mixed monolayers and the surface pressure under compression. At low mole fraction of CUR (*X*_CUR_ = 0.2), the interactions between like molecules (DPPC-DPPC and CUR-CUR) were stronger than that between DPPC-CUR. The interaction between DPPC and CUR molecules in the mixed film was more attractive than that between the molecules in their respective one-component monolayers when 0.4 ≤ *X*_CUR_ ≤ 1. The attractive interaction was strongest in the case of *X*_CUR_ = 0.6. The addition of CUR improved the surface compressibility of the mixed monolayers. The two-dimensional phase diagrams and the interaction energies indicated that DPPC and CUR molecules were miscible in the mixed monolayers. The changes of morphology features of the mixed monolayers obtained from AFM images were consistent with the results from other experimental parameters. The study provides important theoretical support and experimental basis for understanding the mechanism of CUR contact with DPPC molecules.

## Figures and Tables

**Table 1:** The percent of condensation (*C*%) caused by CUR molecules as a function of mole fraction of CUR at discrete surface pressures.

*π* (mN/m)	*C*%					
	*X*_CUR_					
	0	0.2	0.4	0.6	0.8	1
5	0	−1.175	13.914	19.648	2.279	0
15	0	−0.117	10.983	23.859	18.925	0
25	0	−1.041	9.143	24.730	21.455	0
35	0	−0.652	9.723	25.922	23.995	0
45	0	−0.333	9.923	26.534	25.437	0
